# Length of stay in asylum centres and mental health in asylum seekers: a retrospective study from Denmark

**DOI:** 10.1186/1471-2458-7-288

**Published:** 2007-10-11

**Authors:** Peter Hallas, Anne R Hansen, Mia A Stæhr, Ebbe Munk-Andersen, Henrik L Jorgensen

**Affiliations:** 1 Højdevangs Allé 9 st, 2300 Copenhagen, Denmark; 2 National Institute of Public Health, Øster Farimagsgade 5, 1399 Copenhagen, Denmark; 3 Hörda 41, 34014 Lagan, Sweden; 4 The Danish Red Cross Asylum Department, Dag Hammarskiölds Allé 28, Box 810, 2100 Copenhagen, Denmark; 5 Department of Clinical Biochemisty, Bispebjerg University Hospital, Bispebjerg Bakke 23, 2400 Copenhagen NV, Denmark

## Abstract

**Background:**

The length of stay in asylum centres is generally mentioned as a possible health risk to asylum seekers. Medical staff working with asylum seekers has claimed that long lengths of stay in asylum centres might cause or aggravate mental disorders. We used records from a large, multiethnic group of asylum seekers to study if the incidence of mental disorders increased with length of stay.

**Methods:**

The study population was asylum seekers in Danish asylum centres run by the Danish Red Cross. General medical care was provided by Red Cross staff who could refer selected cases to medical specialists. If an asylum seeker needed more than three specialist consultations for mental illness or five consultations for physical illness the referrals had to be approved by The Danish Immigration Service. Between July 2001 – December 2002 the Red Cross prospectively registered health related data on all new applications (n = 4516) to the Immigration Service regarding referrals to medical specialists. We used these records to analyse the association between length of stay in the asylum centres and overall rate of referral for mental disorders. Data was analysed using weighted linear regression.

**Results:**

We found that referrals for mental disorders increased with length of stay in asylum centres in a large, multiethnic population of asylum seekers. The association was found in all the categories of psychiatric illness studied and for a majority of the nationality groups studied.

**Conclusion:**

Length of stay in asylum centres was associated with an increase in referrals for mental disorders in a large, multiethnic group of asylum seekers. The present study supports the view that prolonged length of stay in an asylum centre is a risk factor for mental health. The risk of psychiatric illness among asylum seekers should be addressed by political and humanitarian means, giving prevention of illness the highest priority.

## Background

The length of stay in asylum centres is generally mentioned as a possible health risk to asylum seekers. Medical staff working with asylum seekers have claimed that long lengths of stay in asylum centres might cause or aggravate mental disorders.

A study from Denmark using data from 1986–1988 showed an increase in psychiatric illness with length of stay among asylum seekers [1]. No recent study, however, has focused on the effects of length of stay on the mental health on a large, multiethnic group of asylum seekers. The reasons for this are probably that the available information is often not suited for epidemiological analysis and that there are difficulties in establishing reference groups. Recent studies on the association between length of stay and mental disorders have so far focused on small groups, e.g. a specific nationality [2], asylum seekers in detention [3-5] or have focused on a specific diagnosis [6].

We studied records from a large, multiethnic group of asylum seekers to see if the incidence of mental disorders increased with length of stay.

## Methods

The Danish Red Cross Asylum Department takes care of approximately 90% of the total population of asylum seekers coming to Denmark. General medical care is provided by physicians and nurses from the Danish Red Cross. The physicians from The Danish Red Cross can refer asylum seekers for specialist medical treatment according to guidelines from the Danish Immigration Service. A maximum of five consultations for physical illness and three consultations for mental illness requires no additional administrative steps to be taken. But if an asylum seeker needs more than five/three specialist consultations the referrals for the additional consultations have to be approved by The Danish Immigration Service, which provides the funding for these treatments.

From July 2001 till December 2002 the Danish Red Cross registered the health related data on all applications to The Danish Immigration Service regarding referrals to specialist consultations. In the 18-month period there were 4516 new applications regarding referrals to medical specialists. For 3960 (88%) of these a diagnosis was recorded according to the ICD-10 Classification of Mental and Behavioural Disorders. Diagnoses were made by the referring health care professionals at the asylum centres or by a health care professional at The Danish Red Cross Asylum Department. Only one diagnosis was registered on each application. 2152 (54%) of the applications with a diagnosis concerned a mental disorder.

Background data on the total population of asylum seekers in Denmark, provided by The Danish Immigration Service, was used to identify the number of asylum seekers in each time interval. Data for the total population is used for the analyses. In the background data, the length of stay of the asylum seekers was only stated as the time spent in asylum centre every six months while the records from the Red Cross contained information on the date the application was submitted; as a result the background data are slightly skewed compared to the records from the Red Cross.

Applications were grouped in intervals of 90 days according to the length of stay of the asylum seeker at the time of application. The number of asylum seekers naturally fluctuates and the population of asylum seekers in the asylum centres continuously changes; some may stay for years, others only for a few days. To find out "the number of possible applicants" for a given 90 day interval, the asylum seeker arriving on e.g. June 1^st ^2001 and leaving 180 days later would be counted twice (in the 0–90 days interval and in the 91–180 days interval). Thus the total number of observations of asylum seekers in the 18 intervals of 90 days is greater than the total number of asylum seekers in the study period. Distribution of asylum seekers per 90 day intervals during the 18 month study period is shown in table 1.

**Table 1:** Distribution of asylum seekers per 90 days interval during the 18 month study period

**Duration of stay in asylum centres (days)**	**Somatic referrals**	**Psychiatric referrals**	**Asylum seekers**
0–90	156	78	21843
91–180	232	160	21726
181–270	214	188	21836
271–360	214	222	21542
361–450	230	293	18537
451–540	165	228	14589
541–630	117	231	10505
631–720	93	167	8258
721–810	92	120	7231
811–900	88	117	6601
901–990	70	109	5315
991–1080	50	78	4260
1081–1170	34	58	3118
1171–1260	31	54	2298
1261–1350	12	25	1350
1351–1440	7	14	740
1441–1530	3	10	483

Total	1808	2152	170232

Since only a small number of applicants had stayed more than 1530 days this was used as cut-off point.

**Figure 1:**
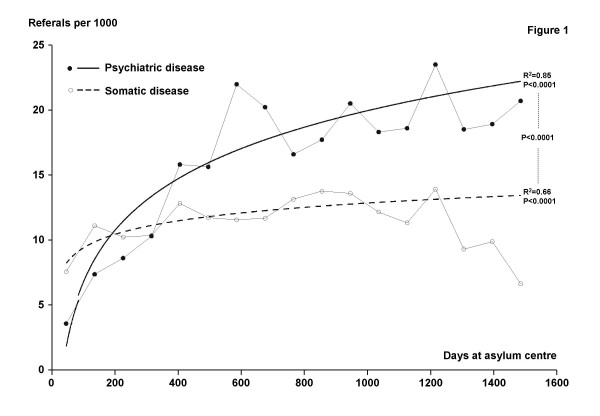
**Length of stay and applications regarding referrals for psychiatric diseases**. Referrals for somatic diseases are shown for reference.

For each time interval, the number of referrals per 1000 asylum seekers, RI, was calculated, and the duration of the stay (in days), DUR, was set as the middle of the interval. The relationship between RI and DUR is not linear (figure 1). The mathematical model best fitting the data was: RI = β*ln(DUR)+intercept. Thus linearized, the total number of asylum seekers in each interval was used as the weight in a weighted linear regression. Parameter estimates, p-values for the model and R-square values were calculated using PROC REG in SAS (Cary, NC, USA).

The p-value for the difference between the slopes for psychiatric disease and somatic disease in figure 1 was calculated using PROC GLM in SAS.

The collection of data for analysis was approved by The Danish Data Protection Agency. This study was retrospective and did not include biological material. Thus according to Danish law no approval from an ethics committee was needed. Due to regulations of The Danish Data Protection Agency, the data had to be formatted for analysis so that all applicants remained anonymous. Therefore it is possible to state only the number of applications, not the number of applicants, and individual asylum seekers could not be linked to individual consultations. As a result, longitudinal regression methods that account for this clustering could not be used.

Since only patients in need of more than 3 consultations for mental disorders or 5 for physical diseases were included, we believe that patients with minor disorders were excluded.

## Results

We found an increase in referrals for mental disorders with increased length of stay in asylum centres in a large, multiethnic population of asylum seekers (fig 1).

Referrals for a psychiatric diagnosis increased significantly with length of stay in the asylum centre: β = 5.7 (95% CI: 4.4; 7.0), Intercept = -19.8 (95% CI: -27.4; -12.3), r^2 ^= 0.85, p < 0.0001.

Referrals for a somatic diagnosis also increased with length of stay: β = 1.49 (95% CI: 0.89; 2.08), Intercept = 2.14 (95% CI: -1.29; 5.57, r^2 ^= 0.66, p < 0.0001.

**Figure 2:**
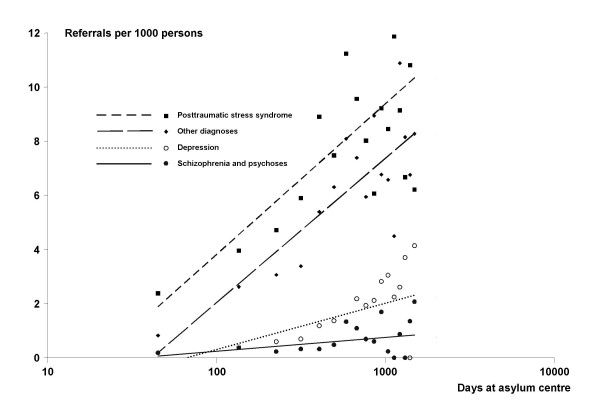
**Categories of psychiatric diagnoses and length of stay in asylum centre**. There was a significant rise in all four categories of psychiatric diagnoses (see table 2).

The distribution of psychiatric diagnosis and the development in referrals for specific categories of diagnosis is shown in table 2 and fig 2.

**Table 2:** Psychiatric diagnoses: distribution of referrals and association with length of stay in asylum centre

IDC-10	Diagnoses	%	β	r ^2^	p
F20.0 – 25.9	Schizophrenia, psychoses	3.8 (n= 83)	0.22	0.28	0.03
F32.0 – 33.9	Depression	9.0 (n = 191)	0.74	0.72	<0.0001
F43.1	Post-traumatic stress disorder	50.8 (n = 1100)	2.42	0.73	<0.0001
Other F	Other psychiatric disorders	36.4 (n = 778)	2.32	0.80	<0.0001
Sum		100 (n = 2152)			

Referrals for mental illness increased significantly with length of stay in the asylum centre within a majority of the nationality groups studied (table 2).

Length of stay versus referrals for mental illness within nationality groups was analysed (table 3).

**Table 3:** Referral rate versus length of stay for different nationality groups

Origin	% of all asylum seekers*	Referrals for psychiatric/psychological treatment		
		
		β	r^2^	p
Bosnia-Herzegovina	10.9	13.1	0.67	<0.0001
Kosovo	11.5	12.0	0.58	0.0004
Other former Yugoslavia	23.7	0.8	0.08	0.3
Iraq	16.4	3.3	0.51	<0.0003
Afghanistan	8.4	2.2	0.10	0.2
Iran	3.8	7.7	0,46	0.003
Middle East, other	8.1	1.7	0.14	0.1
Former USSR	6.1	4.4	0.28	0.03
Other	10.9	4.0	0.61	<0.0001
Sum	99.8			

*(n = 170232)

Refugees from former Yugoslavia (Bosnia-Herzegovina, Kosovo and "Other former Yugoslavia") was the biggest group of asylum seekers in the population studied (table 3). The steepest rise (β) in referrals for psychiatric/psychological treatment was seen for asylum seekers from Kosovo and Bosnia-Herzegovina (table 3).

## Discussion

We found that the need for psychiatric treatment for asylum seekers increased with length of stay in asylum centres, when the association was studied using administrative records on applications for referrals to further specialist treatment. This association was found for all the categories of psychiatric illness studied (table 2) and for a majority of the nationality groups studied (table 3).

Others have studied smaller groups of refugees and asylum seekers and have found similar results: Sack et al. showed an association between depression and recent stressful events in Cambodian refugees [7] and a study of refugees from Kosovo [8] showed that the rate of symptoms of depression increased over time, probably due to postmigration stress factors.

Laban found that the duration of the asylum procedure was an important risk factor for psychopathology among Iraqi asylum seekers with the same level of PTSD [2].

A possible confounding factor in this study could be that the asylum seekers with mental disorders might stay behind in the centres while the healthy are granted asylum or are repatriated. But this does not seem to be the case in other studies of asylum seekers [2,8]. Moreover a study of the asylum system in Denmark, focusing on children with mental health problems, found that the residence case work resembled a random process: There was no difference in the mental health between the groups of children who did or did not obtain asylum in Denmark [9]. Thus a selection process is not a likely explanation of our findings.

The incidence of PTSD rises with length of stay. This could be delayed onset PTSD, but is more likely the consequence of ongoing stress with co-morbid disorders such as anxiety and depression. The possibility of medicalization of social problems should also be taken into account when considered the findings in this study.

Epidemiological analysis based on records of administrative procedures may be confounded: Individual asylum seekers could not be linked to individual consultations, and as a result, longitudinal regression methods that account for this clustering could not be used; this omission affects the standard error estimates. The criteria for declaring a somatic disease required 5 consultations but only 3 were required for mental disorders. This affects the comparison of the level of incidence of psychiatric referrals and somatic referrals.

Finally, the available background data was slightly skewed compared to the records from the Red Cross; thus there is a systematical deviation that might hide significant associations between length of stay and referrals.

**Figure 3:**
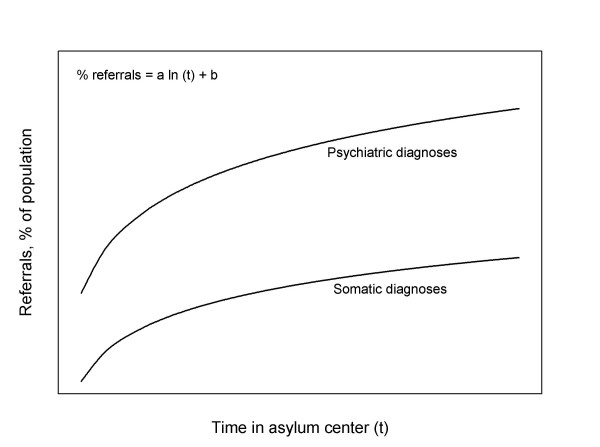
Model for the relationship between length of stay in asylum centres and morbidity.

Our findings can be summarized in a model for the relationship between length of stay in asylum centres and morbidity (fig. 3)

Psychopathology among refugees and asylum seekers is not an inevitable consequence of acute wartime stress but rather reflects the economic, social, and cultural conditions from which they have escaped and into which they are placed [10,11]. Thus the model illustrates how the incidence of referrals for psychiatric illness can be reduced:

i) Reducing length of stay in asylum centres will decrease incidence of referrals for psychiatric illness

ii) Initiatives focusing on improving the mental well-being of asylum seekers could decrease the incidence of referrals for psychiatric illness (shifting the curve to the right).

A previous study by Kjersem [1] has focused on asylum seekers in Denmark. In his study, visits to the general practitioner in the asylum centre increased with time during the first year in the asylum centre. Thus the association between health care use and length of stay is observed in both parts of the administrative system: the general practitioner and as well the specialist. Kjersem theorized the initial steep rise in morbidity during the first year may reflect the asylum seekers improved access to a health care system that had not been available before arrival to the host country. This phenomenon is also seen in fig 3.

The present study substantiates the claim by health care workers that prolonged length of stay in an asylum centre is a risk factor for mental health.

## Conclusion

Length of stay in asylum centres was associated with an increase in referrals for mental illnesses in large, multiethnic group of asylum seekers. The association was found in all the categories of psychiatric illness studied and for a majority of the nationality groups studied.

The risk of psychiatric illness among asylum seekers should be addressed by political and humanitarian means, giving prevention of illness the highest priority.

## Competing interests

The author(s) declare that they have no competing interests.

## Authors' contributions

PH conceived of the study, analysed data and drafted the manuscript. ARH participated in the design of the study, collected information and helped draft the manuscript. MAS organised design of application forms for specialist consultations and organised the registration of data, participated in the design of the study and helped draft the manuscript. EMA participated in the coordination of the study and helped to draft the manuscript. HLJ did the statistical analysis and helped to draft the manuscript. All authors read and approved the final manuscript.

## Pre-publication history

The pre-publication history for this paper can be accessed here:


